# Riding with the flow: study on intrinsic motivation, dispositional flow, and sensation seeking among Italian motorcycle racers

**DOI:** 10.3389/fspor.2025.1501123

**Published:** 2025-06-13

**Authors:** Paolo Mancin, Maria Stefania Ionel, Silvia Cerea, Eleonora F. M. Riva, Martina Rapisarda, Marta Ghisi

**Affiliations:** ^1^ Department of General Psychology, University of Padova, Padova, Italy; ^2^ Sport and Exercise Psychology Group, RIDDLE Lab, Department of Psychology, Babes-Bolyai University, Cluj-Napoca, Romania; ^3^ Department of Biomedical Sciences, University of Padova, Padova, Italy; ^4^ Department of Cultural Heritage and Environment, University of Milano, Milano, Italy; ^5^ U.O.C. Hospital Psychology, University-Hospital of Padova, Padova, Italy

**Keywords:** motorcycle racer, motorsport, intrinsic motivation, flow, sensation seeking

## Abstract

**Background:**

There is a limited body of research investigating psychological dimensions involved in motorcycle racing. Exploring intrinsic motivation becomes crucial since it is associated with high performance and sport persistence. Dispositional flow and sensation seeking, psychological traits involved in motorcycle practice, may exert a significant influence on intrinsic motivation. The current study aimed (1) to assess whether higher levels of intrinsic motivation characterize motorcycle racers participating in higher levels of competition, while no clear hypotheses were made for dispositional flow and sensation seeking, and (2) to assess how dispositional flow and sensation seeking would relate with intrinsic motivation in a sample of Italian motorcycle racers.

**Method:**

Data was collected from 75 motorcycle racers, self-identified as men (age 39.44 ± 13.07 years), registered in the Italian Federation of Motorcycling: 43 competed at National/International level and 32 at Regional level as their highest level.

**Results:**

Motorcycle racers competing at Regional level and National/International level did not differ in psychological dimensions. Moreover, dispositional flow was positively associated with all dimensions of intrinsic motivation, while sensation seeking was associated only with intrinsic motivation to stimulate. Finally, intrinsic motivation to stimulate was associated with both psychological dimensions within a hierarchical regression model.

**Conclusion:**

These findings highlighted that motorcycle racing could be intrinsically rewarding and could enable riders to experience flow and to reach intense emotions regardless of the level of the competition. Furthermore, dispositional flow emerged as significantly associated with intrinsic motivation to sport practice in motorcycle racers, while sensation seeking played a marginal role.

## 1 Introduction

Motorcycle racing is a sport that requires the athlete to interact with a vehicle and a specific track. It comprises multiple disciplines (e.g., motocross, enduro and motorally, trial, speedway, supermoto or motard, and road racing) that are distinguished by specific requirements of the motorcycle and characteristics of the track (e.g., street legal or speed-based motorcycle; natural terrain or asphalt-based surface) (1, 2).

Most studies on motorcycle racing focused on bike functions and human interaction with it. Consequently, there is a paucity of studies focusing on psychological variables (3). Addressing this gap would enable scholars and practitioners to better understand the characteristics of this sport and how to improve the performance of these athletes.

Among the psychological dimensions involved in sport practice, motivation and, more specifically, intrinsic motivation may have important implications in motorcycle racing. Drawing from the Self-Determination Model, intrinsic motivation can be defined as engaging in an activity for the inherent pleasure and satisfaction it provides from being involved in it, and could be placed within a continuum that includes other forms of motivations, namely, extrinsic motivation (i.e., being motivated by external feedback or rewards) and amotivation (i.e., the absence of motivation) (4–6). Intrinsic motivation is also categorized into three different forms: intrinsic motivation to know (i.e., motivation through learning, exploring, or trying to understand something new), to accomplish (i.e., motivated to accomplish or create something), and to stimulate (i.e., motivated to experience fun, excitement, and stimulation) (7, 8). Intrinsic motivation appears as fundamental in sport practice since it is associated with persistence in sport practice (9), with sport commitment (such as enthusiastic commitment) (10), and with high performance in athletes of various disciplines (11, 12). Relevant finding from previous research indicates that athletes with high levels of intrinsic motivation demonstrate a greater likelihood of achieving higher performance levels in individual sports, whereas athletes characterized by fear of failure exhibit a decreased likelihood (13). Within the field of motorcycle racing, intrinsic motivation could be associated with other psychological dimensions, such as flow and sensation seeking.

Flow could be defined as a psychological and physiological state, highly pleasant and rewarding, associated with complete involvement in the activity performed and the sensation of “clicking into place” (14). According to its definition, flow and intrinsic motivation are often considered as two strongly associated constructs (15): Indeed, the former has been conceptualized as an intrinsically rewarding experience for athletes (16). In addition, these two constructs were found to be positively correlated among motorcycle road racers (17). Flow is mainly considered to be a state dimension, contingent on a specific situation. However, the dispositional flow is also described as the predisposition to experience flow, measured by how frequently it occurs to people in a particular domain (e.g., sport performance in motorcycling) (18). There are different models of flow, but the most referred one involves prerequisites and characteristics of flow (18). The prerequisites include challenge-skills balance (i.e., situations that are perceived as challenging by the athletes, and that require extending beyond typical capabilities), clear goals about the task to perform, and unambiguous feedback, that notify the athletes about the rightness of their progress toward the goal. Characteristics that describe what the athlete experiences during flow include total concentration on the task at hand (i.e., complete focus on the task and avoidance of internal or external distractions), action-awareness merging (i.e., feeling at one with the task performed), sense of control (i.e., feeling of being in total control of the task performed), loss of consciousness (i.e., decreased sense of self- and social evaluation), and transformation of time (i.e., experiencing time as going faster or slower than usual) (18). Finally, the last characteristic of flow is autotelic experience, which refers to flow as an intrinsically rewarding experience (18). As described for intrinsic motivation, there is a positive association between flow and high levels of performance (19–22). Thus, flow could be a relevant experience in sport performance among motorcycle racers too.

Lastly, sensation seeking, characterized by the desire to experience novel and thrilling sensations with a willingness to accept potential physical, financial, and social risks solely for the sake of the experience itself (23), may also play a role in motorcycle practice. It is noteworthy that motorcycle racing is frequently classified as an extreme/high-risk sport (24). Extreme sports are defined as “independent adventure activities where a mismanaged mistake or accident is most likely to result in death” (25). They typically involve competitive activities that expose participants to unconventional physical and mental challenges such as speed, height, depth, or natural forces (24), elements that are commonly encountered in various motorcycle disciplines. For example, motocross involves high speed competitions, which take place on natural terrains characterized by dirt, sharp turns, high hills. Enduro competitions could take place on offroad tracks (mule tracks, country roads) and require the racer to face unexpected obstacles and unknown routes (1). J. Olivera Betrán and A. Olivera Betrán (26) proposed a revised taxonomy for extreme sports, classifying motocross, enduro, or rallies under the category of ‘fire wheeled engines’. A meta-analysis examining the relation between personality traits and participation in high-risk sports revealed that sensation seeking is not just the most investigated trait but also has a large effect size (Hedges’ *g* = 0.80, *p* < .001) associated with participation in such sports (27). Moreover, several findings have supported the hypothesis that sensation seeking might play a significant role in intrinsically motivating motorcycle racing athletes. Notably, studies have revealed that non-competitive motorcycle riders reported recreation and sensation seeking as primary motivations for practicing this activity (28). Furthermore, previous research has indicated that sensation seeking, including its dimension of thrill seeking, emerged as a predictor for engagement in extreme sports (29) and motorsport involvement (30). Accordingly, individuals involved in high-risk sports (i.e., surfing) demonstrated high levels of both sensation seeking and intrinsic motivation when compared to those involved in low-risk sports (i.e., golfing) (31). Alternatively, the impact of sensation seeking on motivation may exhibit similarities to the potential influence of flow in motorcycle racers. In fact, Schüler and Pfenninger (32) have suggested that sensation seeking and flow might overlap due to their shared pursuit of extraordinary emotions, as reflected in their respective definitions. In accordance with this notion, flow has been identified as a significant predictor of underestimation of risk perception among kayakers (i.e., another extreme sport) (32) and has demonstrated a positive influence on speeding behaviors among riders of heavy motorcycles in Taiwan (33).

The current body of research on motorcycle racing is notably limited, particularly in terms of investigating the interplay between psychological variables involved in this sport. Moreover, existing studies that have explored this topic have predominantly focused on non-competitive motorcyclists (28, 33–35); thus, rendering impossible the generalizability of findings to motorcycle athletes. Consequently, there is a distinct need for additional studies that specifically examine the psychological dynamics of motorcycle racing while considering the unique attributes of competitive motorcycle athletes.

Furthermore, there is a significant gap in the literature regarding the examination of intrinsic motivation among motorcycle racers and its association with flow and sensation seeking. To address this gap, the present study aimed to compare motorcycle racers from various disciplines, categorized according to their highest self-reported level of competitions performed, in terms of intrinsic motivation, dispositional flow experienced during motorcycling, and sensation seeking. Secondly, the study aimed to explore how dispositional flow and sensation seeking would be associated with intrinsic motivation. The hypotheses can be outlined as follows:

*H1*: individuals performing at the National/International level in motorcycle racing were expected to exhibit higher levels of intrinsic motivation compared to those at the Regional level.

Based on previous research highlighting that intrinsic motivation reinforces persistence in sport practice in various disciplines (9, 13), it can be inferred that intrinsic motivation could characterize engagement at a high level of competition in motorcycle racing. Indeed, Regional level competitions, common starting point for motorcycle racers interested in this sport, are generally more accessible, take place at local circuits with easier tracks and shorter durations, receive minimal media coverage, and are commonly self-funded by participants. Conversely, National and International competitions are more demanding, take place in specific locations with advanced circuits and longer durations, receive greater media coverage, and require greater financial support. Hence, motorcycle racers participating in these competitions are usually more skilled and require a team and staff supporting them preparing for the competition (36).

No specific hypotheses were formulated regarding the associations of flow and sensation seeking with levels of competition in this study.

*H2*: both dispositional flow and sensation seeking were expected to predict levels of intrinsic motivation in motorcycle racers equally.

Consistent with previous findings (17, 29, 30, 32, 33), it was hypothesized that both dispositional flow and sensation seeking should emerge as significantly associated with engagement in motorcycle practice. However, it remained unknown whether these factors hold equal relevance when considered together and whether their associations are similar among motorcycle athletes, as no previous studies have specifically addressed these aspects.

By undertaking this study, we aimed to better understand the psychological dimensions involved in motorcycle racing and their interrelations. Additionally, we made a first attempt in understanding whether psychological models applied in other individual sports were also applicable to motorcycle racing. Finally, this research could contribute to understand the psychological variables associated with motorcycle racing and how to foster persistence in motorcycle sport practice.

## 2 Materials and methods

### 2.1 Participants

In determining the sample size needed to detect medium effect size, we conducted an *a-priori* power analysis using *G-Power* software (version 3.1), and followed the approach of Faul et al. (37). Our analysis indicated that the total number of participants needed for detecting such effect (*ƒ*^2^ = .15), for a linear multiple regression that includes two predictors, with a power of.80, was 68 participants.

We collected data from 75 motorcycle racers (43 competed at National/International level and 32 Regional level as their highest level) who were registered in the Italian Federation of Motorcycling, self-identified as competitors, and volunteered to respond to our invitation (see Procedure). All of them self-identified as men. The sample included ages between 18 and 65 (*M* = 39.44, *SD* = 13.07), and education ranged between 8 and 18 years (*M* = 13.77; *SD* = 2.90). Experience in motorcycling ranged between 6 and 540 months (*M* = 199.07, *SD* = 135.82). The different disciplines of motorcycle racing the participants were involved in included: enduro (36%), motocross (33.3%), velocity (road racing) (24%), trial (2.7%), supermoto (1.3%), speedway (1.3%), and motorally (1.3%). Regarding marital status, 17.3% of participants were single, 26.7% were in a relationship, 42.7% were married or cohabitants, and 9.3% were separated or divorced. As for current occupation, 60 participants had a job (80%), 4 participants (5.3%) were students, and the rest did not identify in the previous categories.

### 2.2 Measures

A brief informative form was used to assess socio-demographic (gender, age, education, marital status, occupation) and sport-related (experience in motorcycling, the highest level of competition performed, type of motorcycle sport practiced) information of each participant.

*Sport Motivation Scale* (SMS) (7, 8) assesses individuals’ level of motivation towards sport. The seven factors (three types of intrinsic motivation, four types of extrinsic motivation, and amotivation) were measured with 28 items rated on a 7-point Likert scale from 1 (*does not correspond at all*) to 7 (*corresponds completely*). For the purposes of the study, we decided to use only the three subscales that assess intrinsic motivation. Internal consistencies for the three selected factors were (Cronbach’s alpha): *α* = .86 for SMS – *intrinsic to know*, *α* = .78 for SMS – *intrinsic to accomplish*, *α* = .68 for SMS – *intrinsic to stimulate*.

*Dispositional Flow Scale-2* (DFS-2) (38, 39) measures an individual’s predisposition to experience flow as a stable characteristic or a trait of their personality. Since participants are required to think about a general episode in which they experienced flow, we specifically asked individuals to reflect on an episode related to motorcycle practice. The nine factors were measured with 36 items rated on a 5-point Likert scale from 1 (*never*) to 5 (*always*). For the objective of the study, only the total DFS-2 score was selected; it showed excellent internal consistency (Cronbach’s *α* = .94).

*Brief Sensation Seeking Scale* (BSSS) (40, 41) assesses the personality trait based on seeking new, complex, and intense sensations and the risk of experiencing them. The four dimensions (*experience seeking*, *boredom susceptibility*, *thrill and adventure seeking*, *disinhibition*) were measured with 8 items rated on a 5-point scale from 1 (*strongly disagree*) to 5 (*strongly agree*). Overall, the BSSS allows to compute a general score of sensation seeking, that was ultimately utilized for the analyses. Internal consistency reliability as measured with Cronbach’s alpha was .57.

### 2.3 Procedure

Participants affiliated with the Italian Federation of Motorcycling were recruited through a newsletter sent to all enrolled members. To be eligible for the study, participants needed to be at least 18 years old and to be motorcycle racers. Each participant received an online link consisting of an informed consent, a socio-demographic information schedule, and a battery of self-report questionnaires. The informed consent instructed participants about the purposes of the study, the voluntary nature of their participation, and the possibility to withdraw from the study at any time without penalty.

The study was conducted in accordance with the Declaration of Helsinki and approved by the Ethics Committee of Psychological Research of the University of Padova.

### 2.4 Data analysis

All the statistical analyses were performed with SPSS, version 27.0 for Windows. First of all, we estimated the descriptive statistics (means and standard deviations for all the measured variables); then, a t-test was conducted to compare motorcycle racers that performed at Regional level and at National/International level. Cohen’s *d* was used to assess the effect sizes and are considered as small (*d* = .20), medium (*d* = .50), and large (*d* = .80) (42). Finally, based on correlational analyses, we employed hierarchical regression, utilizing the SMS scores as dependent variables and the DFS-2 total score and the BSSS total score as independent variables.

## 3 Results

### 3.1 Group comparisons between motorcycle racers

After performing a *t*-test, differences in age, motorcycle expertise (expressed in months), intrinsic motivation to know, to experience, and to accomplish (the scores at three subscales of the SMS), dispositional flow (the DFS-2 total score), and sensation seeking (the BSSS total score) did not emerge as significant between motorcycle racers that competed at Regional level (*n* = 32) and at National/International level (*n* = 43) (Table 1).

**Table 1:** Comparisons between motorcycle racers that compete at regional level and national/international level on age, years of motorcycle expertise, intrinsic motivation, dispositional flow, and sensation seeking.

Variable	Regional level (*N* = 32) M (SD)	National/international level (*N* = 43) M (SD)	*t*_(73)_	*p*	*d*
Age	37.34 (12.75)	41.00 (13.23)	−1.20	.23	−.28
Years of motorcycle practice (in months)	117.25 (122.56)	215.30 (144.16)	−1.20	.23	−.28
SMS—to know	21.41 (5.62)	20.33 (5.34)	.85	.40	.20
SMS—to accomplish	23.41 (3.82)	23.88 (3.75)	−.54	.59	−.13
SMS—to stimulate	25.47 (2.70)	25.40 (3.02)	.11	.91	.03
DFS-2 total score	3.72 (.60)	3.86 (.57)	−.97	.33	−.23
BSSS total score	26.88 (4.61)	25.30 (4.98)	1.40	.17	.33

Years of motorcycle expertise were expressed in months.

SMS—to know, Sport Motivation Scale—intrinsic motivation to know; SMS—to accomplish, Sport Motivation Scale—intrinsic motivation to accomplish; SMS—to stimulate, Sport Motivation Scale—intrinsic motivation to stimulate; DFS-2, Dispositional Flow Scale—2nd edition; BSSS, Brief Sensation Seeking Scale.

### 3.2 Associations among intrinsic motivation, dispositional flow, and sensation seeking

Since motorcycle racers did not differ on the above-mentioned variables, we considered the whole sample for further analyses (*n* = 75). As shown in Table 2, the subscales “intrinsic motivation to know” and “intrinsic motivation to accomplish” of the SMS were positively associated with dispositional flow (respectively, *r* = .25; *p* = .03; and *r* = .40; *p* < .001), but not with sensation seeking (respectively, *r* = .12; *p* = .30; and *r* = .18; *p* = .13). On the other hand, the subscale ‘intrinsic motivation to stimulate’ showed a moderate correlation with dispositional flow (*r* = .43; *p* < .001) and a weak correlation with sensation seeking (*r* = .26; *p* = .02).

**Table 2:** Bivariate correlation between age, years of motorcycle expertise, intrinsic motivation, dispositional flow, and sensation seeking.

Variable	1	2	3	4	5	6	7
1. Age	1						
2. Years of motorcycle expertise	.54***	1					
3. SMS—to know	−.05	−.09	1				
4. SMS—to accomplish	−.04	−.08	.63***	1			
5. SMS—to stimulate	−.09	−.17	.28*	.43***	1		
6. DFS-2 total score	.18	−.04	.25*	.40***	.43***	1	
7. BSSS total score	−.20	−.09	.12	.18	.26*	.12	1

* *p* < .05.

*** *p* < .001.

Years of motorcycle expertise were expressed in months.

SMS – to know, Sport Motivation Scale – intrinsic motivation to know; SMS – to accomplish, Sport Motivation Scale – intrinsic motivation to accomplish; SMS – to stimulate, Sport Motivation Scale – intrinsic motivation to stimulate; DFS-2, Dispositional Flow Scale – 2nd edition; BSSS, Brief Sensation Seeking Scale.

As a result, only the latter subscale of the SMS was used as a dependent variable for the hierarchical regression model (Table 3). In Step 1 of the analysis, the inclusion of dispositional flow (the DFS-2 total score) as a predictor yielded a significant positive relation and accounted for 18.4% of the variance in the dependent variable. In Step 2, the addition of sensation seeking (the BSSS total score) resulted in a significant change (*ΔR*^2^ = 4.4; *F* change = 4.07; *p* = .047). In the final model, both independent variables emerged as significant predictors and explained 22.7% of the variance in the subscale ‘intrinsic motivation to stimulate’ of the SMS.

**Table 3:** Hierarchical regression model of dispositional flow and sensation seeking on intrinsic motivation to stimulate.

Steps	Predictors	B	SE	*β*	*t*	*p*	*F*	df
Step 1							16.43***	1.73
	Constant	17.45	1.99		8.77	<.001		
	DFS-2 total score	2.10	.52	.43	4.05	<.001		
Step 2							10.60***	2.72
	Constant	14.70	2.38		6.18	<.001		
	DFS-2 total score	1.97	.51	.40	3.86	<.001		
	BSSS total score	.12	.06	.21	2.02	.047		

*** *p* < .001.

DV, Sport Motivation Scale – intrinsic motivation to stimulate; B, Unstandardized coefficient; SE, Standard error; *β*, standardized coefficient; *t*, *t*-test; *p*, *p*-values; *F*, F-statistic; df, degrees of freedom; DFS-2, Dispositional Flow Scale – 2nd edition; BSSS, Brief Sensation Seeking Scale.

## 4 Discussion

Given the limited research on psychological variables in motorcycle racers (3), the current study aimed to investigate the relations between intrinsic motivation, dispositional flow, and sensation seeking in a sample of Italian motorcycle racers enrolled within the Italian Federation of Motorcycling. First, motorcycle racers who competed at Regional level were compared with motorcycle racers who competed at National/International level, focusing on sociodemographic, sport-related, and psychological variables. Subsequently, the associations between intrinsic motivation, dispositional flow, and sensation seeking were explored.

Concerning the first aim, no significant differences were observed among motorcycle racers. Thus, regardless of their level of competition, they exhibited similar levels of intrinsic motivation in practicing motorcycle racing. Likewise, comparable levels of dispositional flow and sensation seeking were reported. This finding contradicted previous studies (9, 13) that identified higher levels of intrinsic motivation as being relevant for involvement in higher levels of competitions in other sports.

A possible explanation for this discrepancy was that motorcycling itself may be intrinsically rewarding *per se*, regardless of the level of competition in which individuals perform. In other words, the enjoyment and satisfaction derived from the activity of motorcycling may be a strong motivating factor for individuals, regardless of their competitive level. In contrast with other sports such as basketball, soccer, and volleyball (9) or rowing, mountain biking, swimming, alpine skiing, or equestrian vaulting (13), motorcycle racing might require higher levels of intrinsic motivation to persist even at lower levels of competition. This could be attributed to the unique characteristics of motorcycle racing, which carries a higher risk of fatal injuries and accidents (28, 35). The heightened risk associated with motorcycle racing may intensify the need for strong intrinsic motivation to overcome the inherent challenges and dangers of this sport. This explanation could further support why motorcycle racers did not differ on flow and sensation seeking too. Given the lack of a control group, we cannot draw conclusions regarding whether motorcycle athletes exhibited higher levels of these psychological dimensions compared to athletes in other sports or the general population, which would have further corroborated this hypothesis.

Another possible explanation for these findings could be found in how performance levels have been investigated. In the current study, we relied on a self-report item, asking for the highest level of competition performed, while no objective evaluations were included. Thus, participants could have misinterpreted the item. Moreover, it is possible that some respondents may occasionally engage in high level competitions, such as National and International ones, while being mostly involved in Regional level competitions. This hypothesis could be further supported by lack of differences in age and years of experience. Hence, the two groups identified for the analyses may have areas of overlap.

Similarly, intrinsic motivation, flow, and sensation seeking were not addressed during a performance. Existing findings investigated these psychological dimensions during competitions and with a longitudinal design (9, 11). Competing at National and International levels, compared to Regional level, may elicit higher levels of these psychological dimensions in motorcycle racers at the time of the competition. Future studies should explore these psychological dimensions across different levels of competition recruiting participants during competition seasons.

Finally, the small effect sizes detected suggested that statistically significant differences, if present, may be rare and could potentially emerge with a larger sample size. Hence, these two groups may be indeed characterized by small differences on these psychological dimensions, and the current sample size does not enable to identify them.

As for the second aim, we found small-to-moderate associations between the three forms of intrinsic motivation (i.e., to know, to accomplish, and to stimulate) and dispositional flow experienced while motorcycling. However, sensation seeking showed only a weak association with intrinsic motivation to stimulate. Age and years and experience demonstrated significant correlations only among themselves. In the regression model, we found both dispositional flow and sensation seeking to be significantly associated with intrinsic motivation. However, when examining the standardized coefficients, dispositional flow demonstrated a moderate effect, while sensation seeking demonstrated a weak effect. Overall, these findings highlighted that experiencing flow could be an important factor while engaging in motorcycle racing, as it can foster intrinsic motivation and contribute to the enjoyment of the sport (16).

In contrast, seeking extreme emotions and potentially dangerous situations may not be as relevant in motivating motorcycle racers to practice this sport, despite its considered significance in other extreme sports (29). One possible explanation is that individuals who are highly motivated by sensation seeking may be more likely to drop out sooner from motorcycle racing or withdraw from the Italian Federation of Motorcycling. In fact, this psychological trait has been found to predict disruptive behaviors and potentially dangerous conduct while motorcycling (34), which could undermine involvement in motorcycle races by reducing interest in adhering to rules and fair play. The weak relation between sensation seeking and intrinsic motivation could be ultimately beneficial for motorcycle racers, as it may suggest that they could be less inclined to engage in risky behaviors and experiences, possibly increasing injury risk. Previous research has indicated that risk and experience seeking are associated with a higher risk of injuries in individuals involved in extreme sports (29) and linked to risky behaviors in motorcycling (28). Additionally, motorcyclists who engage in frequent dangerous behaviors on the road, such as following a vehicle too closely, driving through amber traffic lights, or dangerous overtaking, are more likely to be involved in street accidents (35).

Notably, flow and sensation seeking explained only 22.7% of variance in the regression model. Despite being possibly influential, these psychological dimensions do not appear to fully explain intrinsic motivation to stimulate. Future studies should consider other variables (e.g., perceived self-efficacy) that could be involved in motivating motorcycle racers in practicing this sport.

Moreover, the cross-sectional nature of the study did not enable to discuss cause-and-effect relations: thus, it is possible that intrinsic motivation might lead motorcycle racers to experience flow while performing or influence their levels of sensation seeking. Future studies should consider employing more elaborate methods, such as longitudinal designs, and include a control group comprising recreational motorcyclists or other athletes. For instance, evaluation of state flow experiences, measured during a competition instead of retrospectively, would clarify how these suggested relations manifest while performing.

In contrast to the conceptual overlap suggested by previous research (32), the present study found no significant relation between flow and sensation seeking in the sample of motorcycle racers. This indicated that these two constructs may be experienced as distinct phenomena among motorcycle racers. While both flow and sensation seeking involve seeking exceptional experiences, their relation in the context of motorcycle racing appears to be non-significant. This finding points to the need for further investigation into the unique experiences and psychological dimensions associated with flow and sensation seeking in the specific context of motorcycle racing.

The findings of the regression analysis raised questions about the classification of motorcycle racing as an extreme sport, suggesting that flow may be more relevant than sensation seeking in defining engagement in this particular sport. These findings also implied that motorcycle racing shares similarities with other individual sports, as evidenced by the association between intrinsic motivation and dispositional flow, even when accounting for sensation seeking. In line with this hypothesis, the current study did not find a significant correlation between flow and sensation seeking in the sample of motorcycle racers, suggesting that these constructs are experienced as distinct phenomena within this population. Although there may be a conceptual overlap between flow and sensation seeking (3), the present findings supported the notion that they represent different experiences among motorcycle racers.

### 4.1 Limitations and future directions

This study had several limitations that should be acknowledged. First, we explicitly asked participants to refer to their experiences of flow in the context of motorcycling while filling in the DFS-2, which may have influenced the associations with intrinsic motivation. In fact, this focused approach may have contributed to stronger associations between the measures. Second, some of the measures used in the study exhibited low internal consistency, which could have impacted on the reliability of the findings. In particular, the BSSS showed low internal consistency (*α* = .57), highlighting the importance of interpreting the findings with caution. To date, the BSSS represents a brief and useful measure to assess sensation seeking; future studies could consider including a different measure to investigate this psychological construct and its associations with intrinsic motivation and flow, as well as competition level. Third, the cross-sectional design of the study and the retrospective investigation of these psychological dimensions prevented the possibility to establish causal relations or determine the direction of the observed associations. As previously mentioned, longitudinal associations should be explored: motorcycle racers may experience variations in their levels of intrinsic motivation, flow, and sensation seeking before, during, and after a competition. Similarly, the associations with intrinsic motivation could change across time: sensation seeking may be influential at the initial stages of motorcycle racing practice, while flow may be more relevant to sustain persistence and continued participation in competitions. Additionally, the absence of a control group, such as a sample of individuals from the general population or recreational motorcyclists, restricted the generalizability of the findings. Fourth, we did not collect objective data pertaining to participating in competitions. As briefly mentioned before, participants may have misinterpreted the item, leading to potentially erroneous classification. Fifth, the sample was composed only by men, limiting the generalizability of the findings to motorcycle racers who self-identifying in other genders (e.g., women, gender non-binary). Sixth, self-selection bias could have influenced participation in the study, allowing responses from highly motivated motorcycle racers only. Finally, even though sample size was adequate according to the power analysis, a larger sample size could provide a more detailed analysis of the individual dimensions of flow, instead of solely relying on the total score, could allow to capture more nuances in the associations among constructs, and could improve the representativeness of the findings. These limitations should be considered when interpreting the results and further research should address these issues to provide a more comprehensive understanding of the psychological dimensions in motorcycle racing.

Future studies in the field of motorcycle racing could consider incorporating an evaluation of racers’ performance to further assess the practical application of current findings. However, assessing performance in this context poses unique challenges, as it involves the complex interaction between human skills and the functioning of the vehicle. Additionally, external factors such as track characteristics, weather conditions, and accidents involving the racer or other participants can significantly impact performance. To address these challenges, experimental studies could be conducted to control potential confounding variables and provide more robust findings. Furthermore, exploring the longitudinal associations between the risks of injuries, flow experiences, and sensation seeking could offer valuable insights into the impact of these psychological variables on the overall well-being of motorcycle racers. This would allow for a better understanding of how these factors interplay over time and potentially influence the racers’ physical and psychological health. Additionally, including a control group would enhance the comparability and generalizability of the findings. This could involve comparing motorcycle racers with individuals engaged in other sports or recreational motorcyclists, providing a broader perspective on the psychological dimensions specific to motorcycle racing. Furthermore, different disciplines of motorcycle racing (e.g., road racing, enduro, motocross) should be considered, exploring different nuances in how intrinsic motivation, flow, and sensation seeking are experienced in different types of motorcycle racers. Addressing these suggestions would contribute to advance the knowledge and understanding of the psychological aspects of motorcycle racing, while also considering the unique challenges and complexities associated with this sport. Moreover, it would also benefit practitioners, who could include intrinsic motivation, flow, or sensation seeking as targets in psychological interventions aimed to improve performance in motorcycle racing.

## 5 Conclusions

To conclude, this study enhanced our understanding of the psychological aspects within motorcycle racing by uncovering the similarities in psychological variables, namely intrinsic motivation, dispositional flow, and sensation seeking, among motorcycle racers participating at different levels of competition. The findings suggested that motorcycle racers, regardless of their competitive level, exhibit comparable levels of intrinsic motivation, while also indicating a significant association between intrinsic motivation and the experience of flow. Conversely, the relation between intrinsic motivation and sensation seeking appeared to be relatively weak. These findings contributed to the existing knowledge base and highlighted the importance of intrinsic motivation and flow in the context of motorcycle racing, with implications for further research and the development of interventions aimed at enhancing racer engagement and performance.

## Data Availability

The raw data supporting the conclusions of this article will be made available by the authors, without undue reservation.
